# Lessons learnt from a three-year pilot field epidemiology training programme

**DOI:** 10.5365/wpsar.2016.7.4.005

**Published:** 2017-09-25

**Authors:** Damian Hoy, A Mark Durand, Thane Hancock, Haley L Cash, Kate Hardie, Beverley Paterson, Yvette Paulino, Paul White, Tony Merritt, Dawn Fitzgibbons, Sameer Vali Gopalani, James Flint, Onofre Edwin A Merilles, Mina Kashiwabara, Viema Biaukula, Christelle Lepers, Yvan Souares, Eric Nilles, Anaseini Batikawai, Sevil Huseynova, Mahomed Patel, Salanieta T Saketa, David Durrheim, Alden Henderson, Adam Roth

**Affiliations:** a Pacific Community, Noumea, New Caledonia.; b University of Sydney, Sydney, Australia.; c Pacific Islands Health Officers’ Association, Honolulu, USA.; d United States Centres for Disease Control and Prevention, Guam.; e University of Newcastle, Newcastle, Australia.; f University of Guam, Guam.; g Epidemiology and Laboratory Capacity Program, Commonwealth of the Northern Mariana Islands.; h Hunter New England Health, Newcastle, Australia.; i Ministry of Health, Republic of Palau.; j World Health Organization, Manila, Philippines.; k World Health Organization, Suva, Fiji.; l Fiji National University, Suva, Fiji.; m World Health Organization, Honiara, Solomon Islands.; n Australian National University, Canberra, Australia.; o University of Hawaii, Honolulu, USA.; p Department of Monitoring and Evaluation, Public Health Agency of Sweden, Solna, Sweden.; q Department of Translational Medicine, Lund University, Malmö, Sweden.

## Abstract

**Problem:**

The Pacific region has widely dispersed populations, limited financial and human resources and a high burden of disease. There is an urgent need to improve the availability, reliability and timeliness of useable health data.

**Context:**

The purpose of this paper is to share lessons learnt from a three-year pilot field epidemiology training programme that was designed to respond to these Pacific health challenges. The pilot programme built on and further developed an existing field epidemiology training programme for Pacific health staff.

**Action:**

The programme was delivered in country by epidemiologists working for Pacific Public Health Surveillance Network partners. The programme consisted of five courses: four one-week classroom-based courses and one field epidemiology project. Sessions were structured so that theoretical understanding was achieved through interaction and reinforced through practical hands-on group activities, case studies and other interactive practical learning methods.

**Outcome:**

As of September 2016, 258 students had commenced the programme. Twenty-six course workshops were delivered and one cohort of students had completed the full five-course programme. The programme proved popular and gained a high level of student engagement.

**Discussion:**

Face-to-face delivery, a low student-to-facilitator ratio, substantial group work and practical exercises were identified as key factors that contributed to the students developing skills and confidence. Close engagement of leaders and the need to quickly evaluate and adapt the curriculum were important lessons, and the collaboration between external partners was considered important for promoting a harmonized approach to health needs in the Pacific.

The Pacific island countries and areas in the WHO Western Pacific Region (the Pacific) are American Samoa, Cook Islands, Fiji, French Polynesia, Guam, Kiribati, Marshall Islands, Federated States of Micronesia, Nauru, New Caledonia, Niue, Commonwealth of the Northern Mariana Islands, Palau, Papua New Guinea, Pitcairn Islands, Samoa, Solomon Islands, Tokelau, Tonga, Tuvalu, Vanuatu, and Wallis and Futuna. The Pacific has widely dispersed populations as well as limited financial and human resources. Health systems are highly reliant on donor funding and are influenced by the development partners’ regional and global health priorities. Despite efforts by different programmes, average life expectancy is generally low (*1*) and has not significantly improved over the past two decades. (*2*) The Global Burden of Disease 2015 Study estimated that lower respiratory infections, ischaemic heart disease, and diabetes cause the greatest disease burden in the Pacific; (*3*) however, due to a scarcity of good-quality useable data, global burden of disease estimates for the Pacific are largely derived from models. (*4*)

There is an urgent need to improve the availability, reliability and timeliness of useable data to better inform, monitor and evaluate actions for halting this triple burden of disease in the Pacific. Substantial amounts of data are often collected in the Pacific, but very little of these are analysed and made available for policy and planning in a timely manner. (*4*) The data that are available show that the Pacific is facing recurrent epidemics of communicable diseases, extremely high rates of noncommunicable diseases (NCDs) (*2*, *5*) and accelerating effects of climate change on health. (*2*)

The purpose of this paper is to share lessons learnt from a pilot field epidemiology training programme, officially known as the Pacific Data for Decision-making (DDM) Programme, or simply, Programme, which was designed to foster informed and appropriate responses to these Pacific health challenges.

## Context

The need for a coordinated and sustainable public health surveillance training programme and the identification of opportunities for field training has been advocated in the Pacific over the past two decades. (*6*, *7*) The Pacific Public Health Surveillance Network (PPHSN) partners, including (in alphabetical order) the Centers for Disease Control and Prevention (CDC), Fiji National University (FNU), Pacific Community (SPC), Pacific Island Health Officers Association (PIHOA) and World Health Organization (WHO), have been building capacity in surveillance and response across the Pacific for many years. (*8*, *9*) Several efforts have been previously initiated to address the gap in Pacific epidemiological capacity, including sending Pacific health staff to Field Epidemiology Training Programmes (FETPs) overseas; however, it was not until 2004 that a harmonized approach to epidemiology training was established in the Pacific itself, as we describe below. Since 2012, one Pacific country, Papua New Guinea, has also established its own successful FETP (Bieb S, et al., unpublished, 2017).

In 2004, the curriculum of the DDM Programme (*10*) of the Fiji School of Medicine and CDC was adapted to the Pacific and delivered in several areas and countries by PPHSN partners between 2004 and 2011. The goal of the DDM Programme was to build capacity in field epidemiology for Pacific health staff whose jobs require them to have a basic understanding of the area but whose skill level needed to be enhanced to perform their responsibilities effectively. The curriculum was focused on surveillance and response to outbreak-prone diseases. Academic accreditation was achieved in 2010 with the establishment of the Post-Graduate Certificate in Field Epidemiology by the Fiji School of Medicine, which is now the College of Medicine, Nursing and Health Sciences of FNU. More recently, meetings of the Pacific Island Health Ministers in 2011 and 2013 reinforced the need to further build epidemiology capacity of staff in the Pacific. Regional development partners were called on once again to assist with training programmes “to address the lack of trained and experienced epidemiologists in the region … [and] development of comprehensive training programmes to develop core competencies in ‘data techs’, ‘epi techs’ and epidemiologists.” (*11*) Further, sound epidemiological capacity was deemed necessary for meeting the obligations of the International Health Regulations (2005) and the WHO Asia Pacific Strategy for Emerging Diseases and Public Health Emergencies. (*12*, *13*)

In response, PPHSN partners revamped the existing DDM Programme to ensure greater student engagement, improve relevance to current Pacific island priorities and needs and adopt a health-system-wide approach applicable to both communicable diseases and NCDs. The modified Pacific DDM Programme, as outlined below, was pilot-tested from 2013 to 2016.

## Action

### Overview of pilot programme

The goal of the Pacific DDM Programme remained unchanged. The main target groups were epi-techs and health workers who must be able to: 1) work with and understand data sets to perform their roles; 2) identify health threats and assure the quality of source data; 3) operate well designed data and surveillance systems; 4) generate, understand, present and explain high-quality information products from these systems; and 5) perform descriptive and basic data analysis. The Pacific DDM Programme consisted of five sequential courses: four delivered as one-week classroom-based courses and one field epidemiology project (**Table 1**). The courses are described in **Appendix I**.

**Table 1:** Courses and number of students completing each course, Pacific DDM Programme, August 2013 to September 2016

Course	Number of times delivered	Number of students undertaking course	Number of students successfully completing course	Percentage of students successfully completing course*
Introduction to Epidemiology and Field Epidemiology	5	112	105	94%
Public Health Surveillance	5	103	94	91%
Outbreak Investigations	12	244	178	73%
Computing for Public Health Practice	2	43	33	77%
Field Epidemiology Project	2	47	26	55%

* Students who did not successfully complete a course either opted out of being assessed or undertook the assessment and did not pass

### Programme entry requirements

Prior to enrolling in the Pacific DDM Programme, students were required to have either a bachelor’s degree or a minimum of five years’ experience in the health sector (demonstrated on their curriculum vitae) with a written and positive reference from a supervisor.

### Teaching methods

The Pacific DDM Programme was delivered in country by epidemiologists working for PPHSN partners, including (in alphabetical order) CDC, FNU, PIHOA, SPC, University of Guam, University of Newcastle (Australia), and WHO. The original DDM Programme was modified to increase the use of participatory learning methods based on adult learning principles. Sessions were structured so that theoretical concepts were presented in an interactive way and reinforced through practical hands-on group activities, case studies and other interactive practical learning methods. On average, each course had six facilitators and 25 students.

### Curriculum

The curriculum accredited previously by FNU was modified substantially. Some objectives were reallocated across the continuum of courses to improve programme flow. Most of the existing presentations, exercises and resources were re-developed to ensure the newly acquired knowledge and skills could be applied immediately by students within their health systems. The Pacific DDM Programme covered both communicable diseases and NCDs. Students assessed the efficiency and effectiveness of their own surveillance systems and developed a plan for strengthening them. Students reviewed and analysed data sets collected at their workplace.

**Fig. 1:**
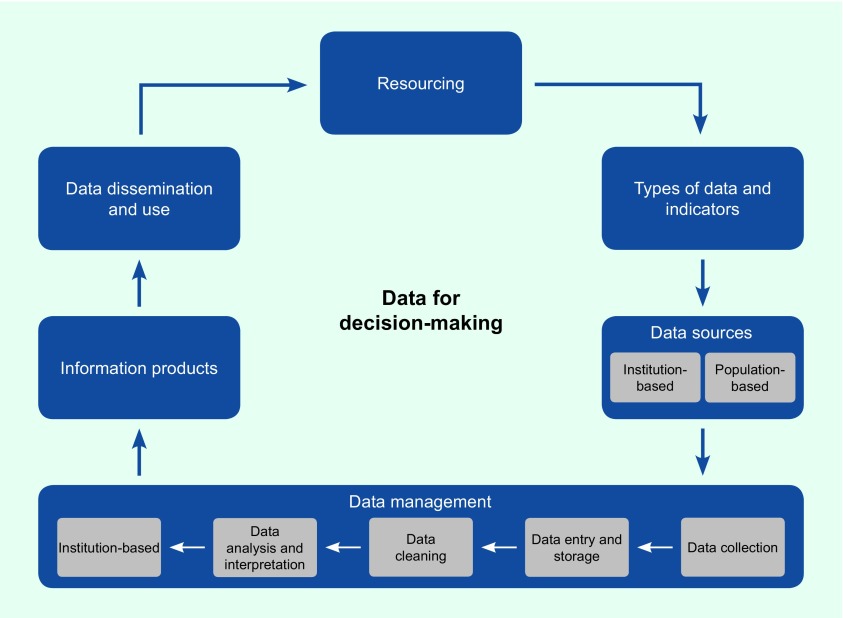
Curriculum framework for Pacific DDM Programme aligned with the Health Metrics Network framework, 2013–2016

In addition, the curriculum was aligned with the Health Metrics Network framework (**Fig. 1**). (*14*) Students were required to develop an information product in each course (e.g. own data analysis product, standard operating procedures for their surveillance systems, outbreak situation reports). The changes to the existing FNU-accredited programme were accredited through an FNU programme amendment.

### Student assessment

Students in each of the first four courses were assessed through both formative and summative assessments. Formative assessments consisted of a variety of assessment methods that did not contribute to the final grade and were intended to provide feedback to students. Summative assessments comprised both continuous (50%) and final endpoint assessment (50%). Continuous assessments consisted of presentation of student products during each classroom-based course. The endpoint assessment was most commonly an exam consisting of multiple choice and short answer questions.

### Monitoring and evaluation

After every classroom-based course, facilitators met to review the course and make necessary refinements. For example, if it was felt that students were not understanding a particular topic, greater time was allocated to it the following day as well as in subsequent delivery of the course. At each course, an evaluation was undertaken to assess students’ self-reported level of understanding and skill pre- and post-course and to capture students’ feedback on the most/least valuable elements and areas for improvement (see **Appendix II**).

A two-day facilitators’ retreat was held at the two-year mark of the pilot phase; focus group discussions were held to review and discuss the pilot phase. The student assessments and standardized course evaluations revealed students’ perceptions of how well the course learning objectives were being met in the short-term. For areas that students found particularly challenging, teaching methods were modified by using more interactive exercises and greater time was allocated to these topics for subsequent course deliveries. Long-term effects on both student competency and their performance in applying the new knowledge and skills in their work setting will require further evaluation.

Further logistical aspects of the Pacific DDM Programme are discussed in **Appendix III**.

## Outcome

From August 2013 to September 2016, 258 students entered into the Programme. Twenty-six course workshops were delivered and one cohort of students completed the full five-course Programme. As of September 2016, 17 students had completed all courses, 32 had completed three courses, 28 had completed two courses and 181 had completed one course. Plans are currently being made to move those students interested through to completion of all courses. The frequency of course delivery was somewhat constrained by the time spent in re-developing the curriculum and funding limitations. Please see **Appendix II** for qualitative findings.

## Discussion

The pilot phase was considered to be highly successful. Student engagement and stakeholder collaboration were considered the two greatest outcomes. Several opportunities for further improvement were also identified.

One of the most significant findings from the pilot phase was the importance of closely engaging Pacific health leaders. At times, leaders had not fully appreciated that the Pacific DDM Programme was a series of sequential courses; consequently, some students were sent to subsequent courses regardless of readiness. Some participants did not have the study and mathematical skills needed to succeed in the courses, which detracted from the learning experience of other students. In the future, before commencing delivery, standardized consultations will be held with health department and other leaders (outlined in **Appendix IV**). Further, the development of self-study “pre-courses” that can be delivered online will be explored.

The facilitators determined that students should begin the Field Epidemiology Project immediately after the first course and be followed up at each of the subsequent courses. This would ensure that the students had more time to complete their project and ensure classroom-based courses were relevant to their projects. Facilitators also considered that there needed to be greater clarity of the specific products for each of the courses. The proposed products of each course are:

1. Introduction to Epidemiology and Field Epidemiology: a clean data set, data dictionary (to be used at the Computing for Public Health Practice course) and data communication brief or infographic;
2. Public Health Surveillance: a planning template for either: (a) CD standard operating procedures (including template for weekly CD surveillance report); (b) an NCD monitoring and surveillance plan (including template for annual NCD reports/dashboard); or (c) standard operating procedures (including report templates) for other routine health information products;
3. Outbreak Investigations: a report on an outbreak investigation (i.e. a situation report);
4. Computing for Public Health Practice: a poster from the data set analysis; and
5. Field Epidemiology Project: (a) CD surveillance standard operating procedures (including weekly CD surveillance reports); (b) NCD disease monitoring and surveillance plan (including annual NCD reports/dashboard); or (c) other routine health information product.

Delivering each DDM course required substantial logistical work (see **Appendix III** for more information). Sustainability will require a dedicated administrative unit to support DDM delivery. One of the greatest challenges of programme implementation was not having a funding stream dedicated specifically to the Pacific DDM Programme. This hampered the ability to plan strategically for the Programme, forecast how many students could be trained and ensure broad coverage across the Pacific. Additionally, some facilitator staff were on short-term contracts and had to pursue other employment at the end of their term. This problem needs to be addressed through longer-term facilitator contracts to minimize staff turn-over and loss of institutional knowledge. This will also help to ensure a high level of course coordination, including consolidated storage and real-time analysis and action from course evaluations. Further, greater contribution of funding and facilitation from countries will help to ensure sustainability.

In recent years, Pacific health ministers urged regional development partners to contribute further to training programmes in epidemiology. The three-year pilot Pacific DDM Programme built on an existing programme and was a direct response to that ministerial request. The Pacific DDM Programme proved popular and achieved high levels of student engagement. The collaboration between external partners was considered important for promoting a harmonized approach to surveillance in the Pacific as was the need for high levels of engagement from Pacific health leaders. The Programme will continue to evolve and adapt to Pacific health needs.
